# Multi-Patient Vision Transformer for Markerless Tumor Motion Forecasting

**DOI:** 10.3390/biomedicines14030496

**Published:** 2026-02-25

**Authors:** Gauthier Rotsart de Hertaing, Dani Manjah, Benoît Macq

**Affiliations:** Institute of Information and Communication Technologies, Electronics and Applied Mathematics (ICTEAM), Université Catholique de Louvain, Place de l’Université 1, 1348 Louvain-la-Neuve, Belgium; dani.manjah@uclouvain.be (D.M.); benoit.macq@uclouvain.be (B.M.)

**Keywords:** lung tumor forecasting, markerless tracking, real-time tumor tracking, vision transformer, digitally reconstructed radiographs

## Abstract

**Background:** Accurate forecasting of lung tumor motion is crucial for precise radiotherapy. Deep-learning-based markerless tracking methods have been explored, but extending these approaches to predict future tumor trajectories remains largely unaddressed. We address this by framing markerless lung tumor motion forecasting as a spatio-temporal prediction task using a vision transformer to estimate three-dimensional tumor positions over short horizons. **Methods:** Digitally reconstructed radiographs (DRRs) generated from four-dimensional computed tomography scans of 12 lung cancer patients were used to train a multi-patient (MP) model. Patient-specific (PS) models trained solely on planning data were compared, and the MP model was further fine-tuned using a small number of patient-specific treatment images under realistic clinical constraints. Models processed sequences of 12 DRRs, with performance evaluated via root mean square error. **Results:** The results indicate that low-resolution inputs with larger patch sizes outperform higher-resolution configurations by reducing image noise. PS models require extensive data to match MP performance, whereas fine-tuning the MP model with limited patient-specific data achieves comparable or superior forecasting accuracy at a lower cost. **Conclusions:** These findings demonstrate that Vision Transformers can extend markerless tracking methods to accurate short-term forecasting and highlight fine-tuning as an efficient strategy for personalized prediction.

## 1. Introduction

Radiotherapy aims to deliver precise and high-dose therapy to tumors while substantially sparing surrounding healthy tissues and critical structures such as the heart and spine. However, its effectiveness depends critically on accurate tumor localization, which is particularly challenging for thoracic and abdominal tumors affected by respiratory motion. Hence, breathing introduces uncertainties in tumor positioning, compelling oncologists to apply safety margins to ensure proper dose delivery, thereby leading to the unwanted exposure of healthy tissues and vital structures [1]. Various techniques have been explored to improve tumor localization and minimize damage to healthy tissues, including breath-holding and abdominal compression [2,3,4,5], as well as respiratory gating [6,7,8]. Nevertheless, breath-holding is not feasible for all patients, and respiratory gating tends to increase treatment time. To overcome these limitations, this paper focuses on a novel real-time tumor tracking (RTTT) method that aims to enable dynamic beam adjustments to the tumor’s position during treatment.

RTTT methods rely on extracting tumor position from X-ray fluoroscopy imaging using either marker-based or markerless approaches. However, because of the inherently low soft-tissue contrast of fluoroscopy images, marker-based tracking methods are often preferred to enhance localization accuracy [1,9]. These methods have been extensively researched and are known for delivering state-of-the-art results in tumor motion forecasting [10,11,12,13,14,15,16,17]. However, they are invasive, requiring surgical procedures that can lead to complications such as pneumothorax and infections [18,19,20,21,22]. They also rely on instantaneous extraction of marker positions and perfect correlation with the tumor centroid, which requires constant updating due to inter-fraction changes. Conversely, markerless methods based on fluoroscopic images are being explored, offering new perspectives for tumor motion management in radiotherapy.

Markerless tumor tracking has been investigated through several approaches, including correlation-based methods exploiting the relationship between tumor motion and diaphragm motion [23,24,25,26,27], as well as template-matching techniques between fluoroscopic images and four-dimensional computed tomography (4DCT) [21,28,29,30]. More recently, advances in machine learning and particularly deep learning have enabled data-driven estimation of tumor motion directly from imaging data, even when the tumor is not directly visible. Most of these approaches rely on architectures such as convolutional neural networks (CNNs) [31,32,33,34,35], convolutional long short-term networks (ConvLSTMs) [36] or U-Net [37]. However, these models primarily address real-time tracking and do not explicitly forecast tumor motion, which is required to compensate for system latency. Indeed, system delays due to acquisition, model prediction, and beam adjustment can reach up to 500 ms [38], which impacts dosimetry and has been shown to reduce clinical target volume coverage [39]. As a result, developing markerless approaches capable of short-term tumor motion forecasting remains an open challenge.

In this paper, we design a markerless real-time tumor tracking approach that explicitly extends instantaneous tracking to short-term tumor motion forecasting. Our learning-based framework leverages a multi-patient pre-trained model, subsequently fine-tuned using a limited amount of patient-specific treatment data, to achieve both generalization and adaptability. The proposed method relies on an encoder–decoder vision transformer (ViT) architecture that exploits spatio-temporal information from sequences of digitally reconstructed radiographs (DRRs) to predict future tumor positions, represented by the tumor center of mass, providing a non-invasive alternative to marker-based forecasting approaches. The main contributions of this work are fourfold:

1. We introduce a novel markerless real-time tumor tracking framework that explicitly addresses short-term tumor motion forecasting to compensate for system latency in radiotherapy delivery;
2. We design an encoder–decoder vision transformer architecture tailored to DRR sequences, highlighting a trade-off between image resolution and temporal context;
3. We provide a quantitative evaluation of multi-patient pre-training and patient-specific strategies, analyzing their data efficiency and robustness across multiple prediction horizons and demonstrating the benefit of multi-patient learning;
4. We release a publicly available GitHub repository (https://github.com/GauthierRotsart/ARIES) enabling reproducible DRR generation and standardized benchmarking of tumor motion forecasting models.

## 2. Materials and Methods

In this section, we describe the dataset, preprocessing steps, model architecture, and training strategies used in this study. We first present the materials, including the 4DCT lung scans and tumor annotations, followed by the methodological details of our markerless tumor trajectory forecasting approach.

### 2.1. Materials

Patients were included according to predefined criteria: (i) confirmed lung cancer diagnosis, (ii) availability of repeated 4DCT scans at two distinct time points, and (iii) availability of associated RTSTRUCT annotations. These criteria resulted in 13 eligible patients from the cancer imaging archive (TCIA) [40]. Each 4DCT volume contains 10 respiratory phases with a slice thickness of 3 mm. For each patient, the gross tumor volume (GTV) was delineated by an expert, and the center of mass of the GTV was used as the tumor position for subsequent analyses. The planning (T1) and treatment (T2) scans were used to simulate patient-specific motion during radiotherapy. All 4DCT volumes were preprocessed to generate DRR images, which served as input to the vision transformer network, following the pipeline described in Section 2.2.2.

### 2.2. Methodology

Our markerless tumor motion forecasting framework is based on an encoder–decoder vision transformer architecture designed to predict future tumor positions from DRR sequences. The network explicitly models spatio-temporal dependencies in the input image sequence to estimate the future trajectory of the tumor center of mass, enabling short-term motion forecasting for real-time radiotherapy applications.

#### 2.2.1. Problem Formulation

In this work, we addressed a spatio-temporal problem: predicting the future trajectory of a tumor’s center of mass from a sequence of DRR images. Let $X=(X_{t-N+1},...,X_{t})$ denote a sequence of *N* past DRR images representing a patient’s radiographic projections, and $Y=(Y_{t+1},...,Y_{t+T})$ the corresponding sequence of future 3D coordinates (x,y,z) of the tumor’s center of mass over a horizon *T*. Our goal is to learn a function f:X→Y, parameterized by a ViT-based network, that predicts autoregressively the entire future trajectory. This task is thus formulated as a sequence-to-sequence regression problem, where the input is a sequence of images and the output is a sequence of continuous 3D vectors representing spatial coordinates.

#### 2.2.2. Data Processing

#### DRRs Generation and Preprocessing

Using the averaged motion model derived from a 4DCT, a patient-specific training dataset was created with tools from OpenTPS [41]. For each patient anatomy, this public software generates a synthetic breathing signal consisting of α points, corresponding to the mean respiratory trajectory extracted from the planning 4DCT, which is then applied to simulate motion. This synthetic breathing signal is applied at the tumor center and combined with the patient’s deformation fields, allowing for the generation of realistic respiratory motion across the entire anatomy. As a result, a sequence of α synthetic intra-fraction 3DCT images is generated. From these volumetric images, DRRs are computed to serve as inputs to the forecasting model. The DRRs are then computed through projection onto the coronal axis. The Beer–Lambert absorption-only model is used to simulate realistic fluoroscopy images. The DRR images were then normalized to values between 0 and 1.

DRR images were first generated to a 512×512 size with spacing of 1 mm × 1 mm. To study the impact of image resolution on the forecasting performance, the DRRs were subsequently rescaled using interpolation to a spatial resolution of W×H, where *W* and *H* denote, respectively, the image width and height in pixels. This preserves the physical field of view during rescaling. Given the ViT-based architecture, the number of tokens processed by the network is given by:

(1) $$N_{tok}=\frac{W\times H}{P^{2}}$$

for a patch size *P*.

In parallel to the image preprocessing steps described above, the tumor motion signal is modeled and normalized. The tumor’s position oscillates with the patient’s breathing and can be expressed as follows:

(2) $$X\left(t\right)=A\left(t\right)+P_{ref}$$

where A(t) represents the breathing-induced displacement around a reference position $P_{ref}$. In practice, the baseline tumor position during treatment may differ from that estimated during planning. Even after rigid registration, a residual baseline shift may persist. To account for this, we assume a maximum possible residual shift of 5 mm in each spatial direction. It is essential to incorporate this baseline shift when defining the normalization bounds: without it, future positions observed during treatment could fall outside the distribution used during training. Therefore, the motion range is defined as $X_{max}=P_{ref}+\left(A_{max}+5\right)$ and $X_{min}=P_{ref}-\left(A_{max}+5\right)$, where $A_{max}$ is the maximum motion amplitude measured during the planning session ($A_{max}=A_{plan}$).

Because the true treatment amplitude $A_{treat}$ is unknown at inference time, normalization must rely on the planning amplitude $A_{plan}$. This ensures that both training and inference operate under consistent and comparable distributions, avoiding a train/test mismatch caused by unobserved differences in motion amplitude. The normalized position is applied independently to each spatial coordinate and thus computed as follows:

(3) $$\hat{X}\left(t\right)=2\frac{X\left(t\right)-X_{min}}{X_{max}-X_{min}}-1$$

#### Training and Test Sets

For each patient, we generated a dedicated training dataset and a separate test dataset. The training dataset was generated from the planning 4DCT anatomy and its associated average respiratory motion, whereas the test dataset was constructed from the treatment 4DCT. The training set consists of 10,000 DRRs, whereas the test set includes 10 free-breathing sequences (20 s each), generated from distinct respiratory trajectories derived from the treatment respiratory signal by adding controlled noise using OpenTPS. These sequences were co-registered to the reference dataset (i.e., the planning session) by performing rigid registration of the mid-position image of each 4DCT, thereby simulating realistic patient setup errors. A fixed frame rate of 5 fps was used, resulting in 1000 images.

#### 2.2.3. Model Architecture and Training

The following section details the ViT-based model and its training procedure.

#### Model Architecture

Vanilla vision transformers [42] are typically employed for classification tasks and consist of a single encoder followed by a classification head. In this work, we used the TrajViViT network [43], which replaces the classification head with a decoder for regression tasks. The encoder’s role is to embed the input images into a feature space, while the decoder autoregressively predicts the motion. Both components comprise six layers, similar to vanilla transformers [44]. Typically, the embedding dimension is 512, and eight attention heads are used. The inner-layer of the feed forward network is composed of 2048 neurons.

Conventional architectures such as CNNs or ConvLSTMs represent input image sequences through hierarchical feature abstractions and typically predict the entire output sequence in a single step, which can limit their ability to capture long-range temporal dependencies. Vision transformers, in contrast, split images into patches and employ a self-attention mechanism to model interactions across all patches and time steps. Moreover, the decoder allows for prediction in an autoregressive fashion, which is particularly useful given the unknown and variable latency in the radiotherapy system. To retain spatial order, a spatio-temporal positional encoding is added to the patch embeddings [42]. Section 3.1 analyzes the dependence of the ViT patch size on the image resolution.

#### Training Setup

Transformers were trained on 25 epochs with a batch size of 12. A cosine annealing scheduler [45] was used, with a one-epoch warmup during which the learning rate was linearly ramped from 1/100 of the peak to its maximum. After the warmup, the learning rate decayed back to its initial value without restarts. The loss function was root mean square error (RMSE) averaged over all forecasted time-points. The Adam optimizer was employed, with Glorot initialization [46]. Teacher forcing [47] was employed to accelerate convergence during training. However, this strategy may introduce a discrepancy between training and inference, as the model is conditioned on ground-truth previous outputs during training but must rely on its own predictions at inference time. Despite this limitation, we observed stable autoregressive behaviour during inference.

#### 2.2.4. Experimental Setup

We first investigated the spatio-temporal properties of the vision transformer, such as the impact of patch size and temporal context, using only a multi-patient (MP) model trained on T2 treatment data with a leave-one-out cross-validation framework. This analysis focused only on design choices related to the model input and representation. Following this analysis, we compared training strategies to assess the benefits of patient-specific planning data. The patient-specific (PS) approach learns individualized features, such as motion amplitude or tissue contrast, from T1 planning data, and was trained on varying amounts of patient-specific data to evaluate its robustness at different prediction horizons, from immediate tracking (T=0) to long-term forecasting (T=10). Additionally, the MP model was fine-tuned on small amounts of patient-specific treatment data using low-rank adaptation [48] to adapt predictions to individual patients for benchmarking evaluation. All strategies used the same vision transformer architecture and input settings, including patch size and sequence length, and were trained independently with consistent hyperparameters (peak learning rates of $5\times 10^{-5}$ for PS models, $1\times 10^{-4}$ for MP models, and a constant $1\times 10^{-2}$ during fine-tuning).

In a final comparison, we compared the proposed encoder–decoder ViT (TrajViViT) with two alternative architectures on two distinct datasets: a ViT encoder-only variant [42] and a ConvLSTM-based tracker [49], both trained under identical training and testing conditions. This comparison was intended to assess the global impact of the autoregressive decoding process under controlled hyperparameter settings, as well as the generalization of the model. However, it does not constitute a strict component-wise ablation study. Although both ViT-based models share the same embedding dimension and encoder configuration, the encoder-only formulation relies on a dedicated CLS token and performs one-shot regression, whereas the encoder–decoder model operates in an autoregressive manner conditioned across temporal steps. Consequently, removing or modifying one of these elements would alter the prediction mechanism rather than isolate a single architectural component.

## 3. Results

In this section, we present the results obtained for the spatio-temporal tumor-trajectory-forecasting task. Unless otherwise specified, all experiments are conducted using a forecasting horizon of one second (T=5) and 10,000 training samples. Although this horizon may exceed the typical latency of clinical real-time tracking systems, it was chosen to systematically characterize the temporal behavior of the proposed models and to analyze performance degradation as the prediction horizon increases. Shorter horizons are evaluated separately. We first investigate the sensitivity of the multi-patient model to key spatial and temporal design choices, namely, the DRR image resolution, the ViT patch size, and the input sequence length. This analysis, conducted exclusively on the MP model due to its offline training capability, allows us to establish a consistent configuration for subsequent experiments.

Using this optimized configuration, we then trained patient-specific models and evaluated their performance as a function of the number of patient-specific training samples. To further characterize temporal robustness, we assessed model accuracy across different prediction horizons, highlighting how performance varies from immediate tracking (T=0) to longer-term forecasting. Finally, we performed fine-tuning of the MP model using small amounts of patient-specific treatment data, comparing the resulting performance with both the MP baseline and an idealized patient-specific baseline (PS-T2, trained with 10,000 patient-specific treatment samples). Additionally, the generalization of the proposed model was assessed on two distinct datasets and compared with two alternative architectures: a ViT encoder-only variant and a ConvLSTM-based tracker. All experiments were repeated over five random seeds to account for variability due to weight initialization.

### 3.1. Spatio-Temporal Analysis

The following spatio-temporal analysis was conducted on a subset of 8 out of the 13 patients. This selection balances computational feasibility with the need to preserve a representative sample while also mitigating the risk of overfitting to the full dataset.

#### 3.1.1. Spatial Dimension

**Figure 1:**
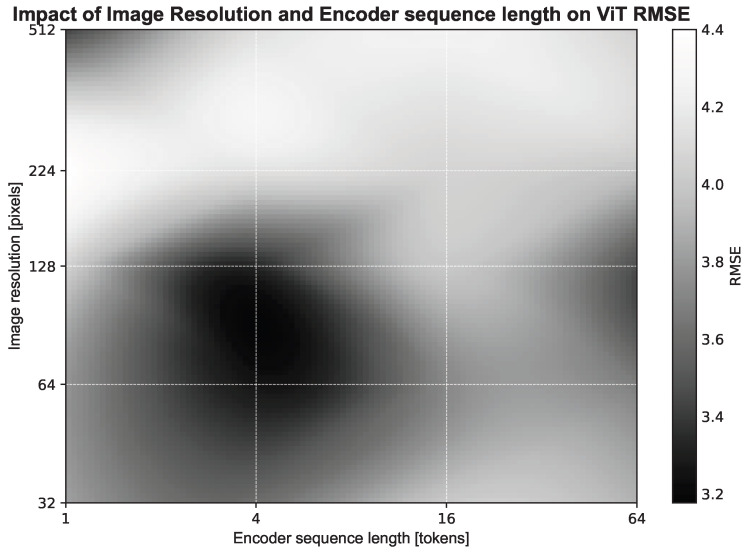
Heatmap showing the RMSE of a Vision Transformer as a function of input image resolution and number of tokens (both axes in log scale). Darker colors indicate lower RMSE, highlighting the combinations of resolution and patch size that yield optimal performance.

We first evaluated the impact of spatial resolution on the forecasting performance of the MP model. Five image sizes were tested on Figure 1: 32 × 32, 64 × 64, 128 × 128, 224 × 224 and 512 × 512. For each resolution, the ViT patch size was adjusted so that the number of tokens fed into the model remains constant, allowing us to probe the effect of spatial granularity while controlling the model’s input dimensionality.

The results show an optimum at a small number of tokens and low resolution, corresponding to a more general representation. A secondary optimum emerges at higher resolutions and larger numbers of tokens, indicating that both resolution and token count influence the ViT’s forecasting performance. There is no statistical difference between the two optima (*p*-value ≥ 0.05). In the remainder of this study, the low-resolution, low-token configuration is retained due to its lower computational cost.

#### 3.1.2. Temporal Dimension

**Figure 2:**
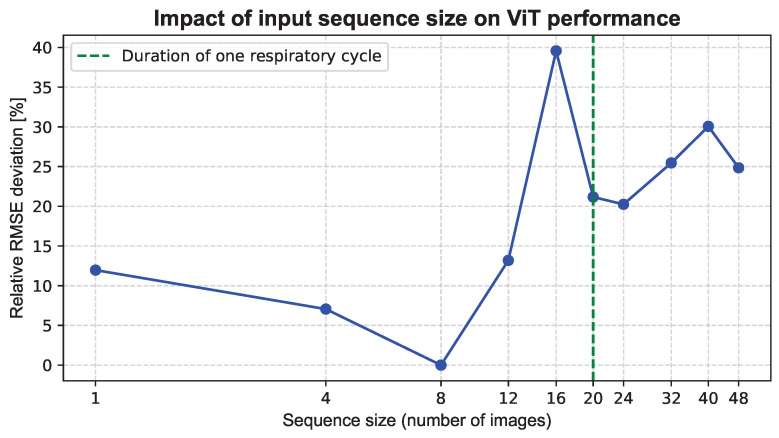
Relative RMSE deviation of the ViT as a function of input sequence size (x-axis in log scale), expressed as the percentage difference with respect to the best-performing configuration (sequence length of 8 images). The vertical line indicates the typical duration of one respiratory cycle (20 images), showing that roughly half a cycle is sufficient for the model to capture the relevant temporal patterns.

We then analyzed the impact of input sequence size on ViT performance using the previously identified optimal spatial configuration (patch size and resolution). The lowest RMSE was observed for a sequence size of eight images. Figure 2 shows the relative RMSE deviation with respect to the best-performing temporal configuration, expressed as the percentage difference relative to the lowest observed RMSE (lower values indicate closer performance to the optimum), highlighting that increasing the number of images beyond 8 does not improve performance. For reference, the typical duration of one respiratory cycle corresponds to approximately 20 images (4 s), indicating that roughly half a breathing cycle is sufficient for the model to capture the relevant temporal patterns.

### 3.2. Training Strategy

While the multi-patient model is trained offline and can therefore be deployed directly at treatment time, it does not explicitly exploit patient-specific information such as individual anatomy or breathing patterns, which may influence the accuracy of short-term tumor motion forecasting. In this section, we investigate whether and under which conditions patient-specific training provides a measurable benefit over a generic MP model while accounting for realistic clinical deployment constraints.

To this end, we compare MP models with PS models trained from scratch, focusing on two complementary aspects: (i) the amount of patient-specific data required to justify model specialization, and (ii) the robustness of patient-specific training across increasing prediction horizons. The deployment cost of PS models is quantified by varying the amount of available patient-specific training data, from 1000 to 25,000 DRRs. The upper limit of 25,000 DRRs corresponds to the maximum amount of data that can be generated and used to train a model between the planning and the first treatment session, amounting to approximately one day of computation on our infrastructure (NVIDIA RTX 6000 GPUs), and reflects a realistic clinical deployment constraint.

#### 3.2.1. Patient-Specific Data

**Figure 3:**
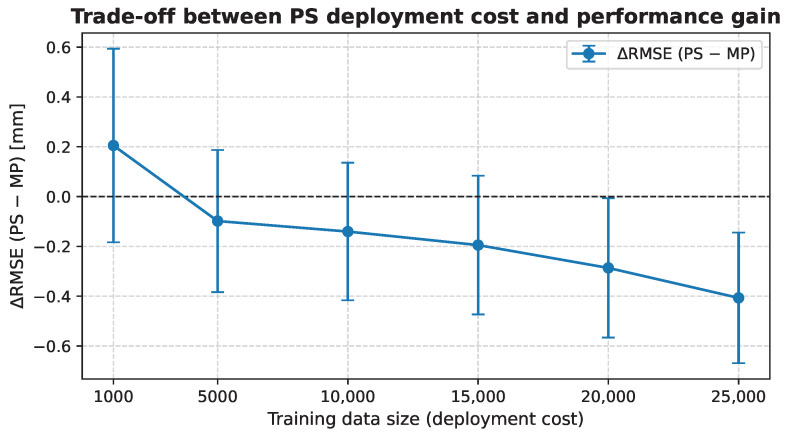
Difference in RMSE between the patient-specific model and the multi-patient baseline as a function of training data size. Negative values indicate improved performance of PS over MP.

Figure 3 compares the performance of patient-specific and multi-patient models using paired evaluations across patients and random seeds at a fixed prediction horizon of T=5. Overall, PS models trained from scratch do not show a statistically significant improvement over the MP baseline (p≥0.05) for low to moderate amounts of patient-specific data. Only when large training sets are available does patient-specific training yield a slight performance gain, indicating that model specialization is costly in terms of data and computation.

#### 3.2.2. Prediction Horizon

**Figure 4:**
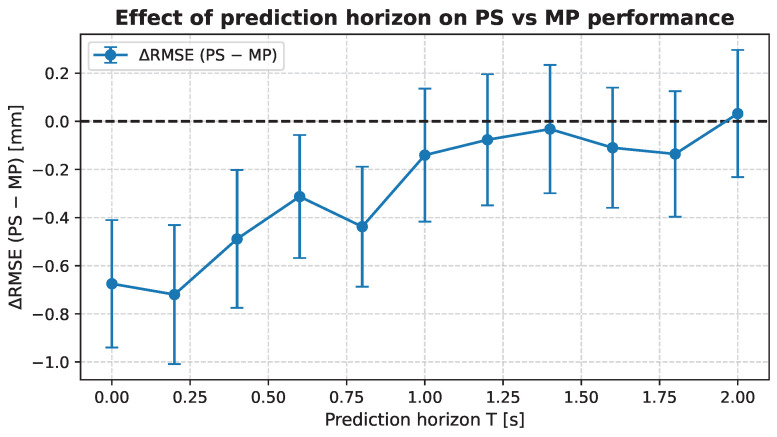
Difference in RMSE between patient-specific and multi-patient models as a function of prediction horizon T (from 0 to 2 s). Horizontal dashed line indicates zero difference.

Figure 4 shows the paired difference in RMSE between patient-specific and multi-patient models for each patient and random seed across prediction horizons from T=0 to T=10. Here, T=0 corresponds to immediate tracking of the current state rather than forecasting future values. For short horizons (T=0 to 4), patient-specific training achieves a statistically significant improvement over the MP model (*p*-value ≤ 0.05). Beyond T=4, no statistically significant difference is observed (*p*-value ≤ 0.05), and the performance gap between PS and MP remains approximately constant. These results indicate that patient-specific models mainly benefit tracking and very short-term prediction, while the MP model exhibits greater robustness as the prediction horizon increases and uncertainty in respiratory dynamics becomes dominant.

#### 3.2.3. Hybrid Strategy: Patient-Specific Fine-Tuning

The previous analyses highlight the limitations of training patient-specific models from scratch in a deployment setting. While such models may provide benefits at short prediction horizons or when large amounts of patient-specific data are available, their training cost and limited robustness at longer horizons restrict their practical applicability. To address these limitations, we investigate a hybrid training strategy based on patient-specific fine-tuning of the pre-trained multi-patient model. This approach assumes that a limited number of annotated patient-specific images become available during treatment and aims to leverage this information without sacrificing the generalization capability of the MP model. For instance, approximate tumor localization may be inferred from cone-beam CT images acquired for patient positioning.

**Figure 5:**
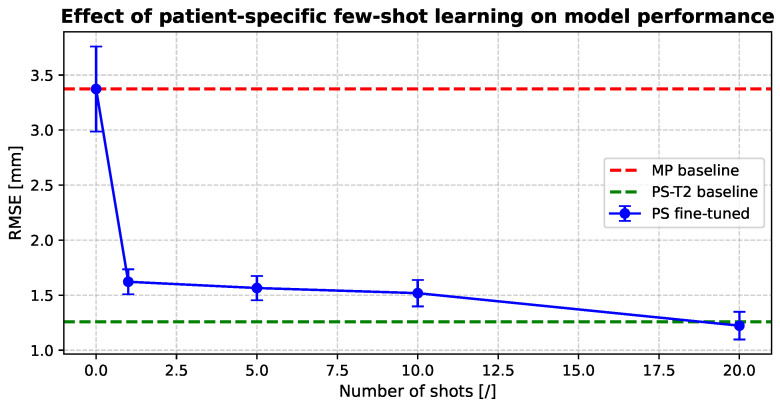
The figure shows that RMSE decreases when the TrajViViT model is locally adapted with patient-specific samples. Two error baselines are included: the red dashed line denotes the MP baseline, and the green dashed line denotes a high-resource (asymptotic) PS-T2 baseline, assuming access to 10,000 patient-specific samples. The results indicate that even a single annotated sample yields an acceptable error level.

Figure 5 illustrates the effect of patient-specific fine-tuning on prediction accuracy when limited annotations (i.e., shots) are available. Performance is compared to the MP baseline (no fine-tuning) and to an idealized patient-specific reference model trained with 10,000 annotated treatment samples. Although this latter scenario is not realistic in clinical practice, it serves as an upper-bound reference to contextualize the performance achieved through fine-tuning. As the number of fine-tuning samples increases, the RMSE consistently decreases, with statistically significant improvements over the MP baseline (p≤0.05). Notably, fine-tuning with as few as 20 annotated treatment images achieves performance comparable to the idealized patient-specific reference, demonstrating that effective patient adaptation can be achieved at a substantially reduced deployment cost.

### 3.3. Generalization Across Datasets

To assess the generalization capability of the proposed approach, we evaluated the multi-patient model trained on the whole TCIA dataset after limited patient-specific fine-tuning. In this setting, the pretrained MP model weights are fixed and directly transferred to two independent datasets: SM (Small Motion dataset) [50] and SV (Small Volume dataset) [51]. These datasets comprise a total of 32 patients collected in the respective studies. For each patient, the thoracic region was acquired using 4DCT with a slice thickness of 2 mm under free-breathing conditions. Audio coaching was used during acquisition to promote regular breathing patterns. The SM dataset contains tumors predominantly located in the upper lung, resulting in relatively small respiration-induced tumor motion. The median breathing amplitude is 2.44 mm, while the gross tumor volume varies from 13 mL to 280 mL. In contrast, the SV dataset contains smaller tumor volumes (median 5.77 mL) and exhibits larger variability in breathing amplitude, ranging from 3 mm to 35 mm.

Table 1 reports the performance of the MP model applied to the SM and SV datasets. This demonstrates that a lightweight adaptation enables effective specialization to new data distributions.

### 3.4. Architecture Comparison

We compared the proposed encoder–decoder ViT (TrajViViT) against a ViT encoder-only variant [42] and a ConvLSTM-based tracker [49], all trained under identical conditions. Table 2 highlights that the encoder–decoder model significantly outperforms both the encoder-only ViT and ConvLSTM baselines when small fine-tuning samples are available (*p* < 0.05), highlighting the advantage of autoregressive decoding in low-data adaptation scenarios. As the fine-tuning budget increases, performance differences narrow and become statistically non-significant (*p* > 0.05), indicating that all architectures eventually converge with sufficient annotated data.

## 4. Discussion

### 4.1. Comparison to Other State-of-the-Art Methods

To the best of our knowledge, most existing markerless approaches primarily address tumor motion tracking rather than explicit forecasting. To enable a meaningful comparison with prior work, we therefore report tracking RMSE performance and restrict the analysis to lung tumor studies that estimate motion in all three clinically relevant directions: superior–inferior, left–right, and anterior–posterior. Reported tracking errors in the literature range from 1.90±0.65 mm, 1.03±0.34 mm, and 1.07±0.35 mm for methods evaluated on small patient cohorts with private 4DCT data (5, 8, and 10 patients, respectively) [31,33,52]. Other approaches have reported tracking RMSE of 1.17, 1.72, and 0.95 mm on the public TCIA dataset [36,53,54], although their DRR generation pipelines are not publicly available. Using the same TCIA dataset, our approach achieves a tracking accuracy of 1.20±0.02 mm after fine-tuning, which is comparable to previously reported results. To facilitate fair benchmarking in future studies, our DRR generation pipeline will be released publicly.

### 4.2. Training Strategies in Clinical Workflow

Our results highlight that training patient-specific models from scratch is costly in terms of data and computation. While PS models can slightly outperform a multi-patient baseline at high data budgets (≥20,000 samples) and short prediction horizons, their robustness decreases for longer horizons where respiratory uncertainty dominates. In contrast, fine-tuning a pre-trained MP model with a limited number of patient-specific images acquired during treatment consistently improves short-term forecasting accuracy. Notably, as few as 20 fine-tuning samples are sufficient to match the performance of a PS model trained from scratch with tens of thousands of samples, demonstrating a practical hybrid strategy that combines the generalization of the MP model with patient-specific adaptation at a substantially reduced deployment cost.

### 4.3. Spatial Resolution and Patch Size of the ViT

The original DRR images have a size of 512 × 512 pixels with a spacing of 1 mm. Reducing the image resolution consequently increases the effective spacing, which directly impacts the precision of the learned representations. As depicted in Figure 1, when keeping the total number of tokens constant, higher image resolution leads the model to focus on fine-grained details, many of which are not relevant for tumor trajectory prediction. Lowering the resolution acts as a form of filtering, reducing the influence of noise present in the DRRs and potentially improving generalization.

However, reducing the resolution while simultaneously using a large patch size limits the spatial granularity of the representations that the multi-patient model can learn. For instance, experiments indicate that a configuration with 128 × 128 resolution and patch size 8 achieves slightly better prediction accuracy (though not statistically significant) than the current optimum of 64 × 64 with patch size 32. This configuration captures finer spatial details but comes at the cost of higher computational demand, as the complexity of the attention mechanism scales quadratically with the number of tokens ($O(N^{2})$).

### 4.4. Limitations and Future Works

While the proposed approach achieves state-of-the-art performance in markerless tumor trajectory forecasting, it relies on the availability of annotated treatment images for patient-specific adaptation or fine-tuning. Moreover, digitally reconstructed radiographs were generated using TomoPy, and a reality gap may exist between these simulated DRRs and actual fluoroscopic projections acquired during treatment, which could potentially affect model performance. In a clinical setting, acquiring such annotations can be costly and time-consuming, which limits the scalability of the method and its deployment in fully automated workflows. Future work should therefore focus on reducing or eliminating the need for annotated patient-specific data. Promising directions include few-shot learning strategies that leverage minimal supervision, as well as zero-shot approaches that aim to generalize across patients without any patient-specific annotations at treatment time. This could involve improved pretraining schemes, domain-invariant representations, or adaptation mechanisms driven solely by unlabeled treatment data. Another important limitation lies in the assumptions regarding patient breathing behavior: our model assumes regular respiratory motion patterns, which may not fully capture the variability observed in actual patients, potentially affecting prediction accuracy. Additionally, incorporating attention heatmaps or saliency maps to improve the interpretability of ViT predictions represents another promising direction for future work, which could enhance clinical trust and understanding of the model’s decisions. Advancing toward these paradigms would further enhance the clinical applicability and robustness of markerless tumor-tracking systems.

## 5. Conclusions

This study is the first to extend the paradigm of markerless real-time tumor tracking to the task of tumor trajectory forecasting using a vision-transformer-based model. Trained on DRR images, the proposed network achieves state-of-the-art performance under multiple deployment scenarios, including both direct deployment of a multi-patient model and patient-specific fine-tuning during treatment.

Beyond prediction accuracy, our results highlight important practical implications for clinical deployment. In particular, training patient-specific models from scratch does not provide a favorable cost–performance trade-off, whereas fine-tuning a pre-trained multi-patient model with a limited amount of patient-specific data enables efficient adaptation without compromising robustness. These findings support the use of markerless, data-driven forecasting approaches as a clinically viable alternative to invasive marker-based strategies. Future work will focus on further reducing the reliance on annotated treatment data by exploring few-shot and zero-shot learning paradigms, with the goal of enabling fully automated and scalable clinical deployment. 

## Figures and Tables

**Table 1:** Generalization of the proposed method on two private datasets. Results are expressed as mean ± standard deviation of RMSE [mm].

FT Data	Dataset	X [mm]	Y [mm]	Z [mm]	RMSE [mm]
1	SM	2.25±0.05	2.48±0.08	1.66±0.08	2.22±0.05
	SV	2.10±0.10	2.30±0.10	3.50±0.14	2.90±0.09
20	SM	1.70±0.002	1.87±0.008	1.21±0.004	1.66±0.004
	SV	1.48±0.02	1.74±0.03	2.76±0.007	2.24±0.01

**Table 2:** Architecture comparison between the proposed method and a vanilla vision transformer and ConvLSTM based trackers for one-shot learning. The results are expressed as mean ± standard deviation of RMSE [mm].

Samples	Models	TCIA	SM	SV
FT = 1	ConvLSTM	1.90±0	2.18±0	3.09±0
	ViT	1.92±0.03	2.45±0.03	3.25±0.03
	TrajViViT	1.62±0.06	2.22±0.05	2.90±0.09
FT = 20	ConvLSTM	1.22±0.01	1.66±0.002	2.27±0.005
	ViT	1.23±0.10	1.61±0.06	2.27±0.008
	TrajViViT	1.22±0.03	1.66±0.004	2.24±0.01

## Data Availability

The public data used in this study are available from The Cancer Imaging Archive (TCIA) at https://www.cancerimagingarchive.net/collection/4d-lung/ (accessed on 21 February 2026). The private data are not publicly available due to privacy and ethical restrictions but may be made available from the corresponding author upon reasonable request and with appropriate ethical approval.

## Abbreviations

The following abbreviations are used in this manuscript:

RTTT	Real-Time Tumor Tracking
CT	Computed Tomography
CNN	Convolutional Neural Network
ConvLSTM	Convolutional Long Short-Term Memory
ViT	Vision Transformer
DRR	Digitally Reconstructed Radiograph
TCIA	The Cancer Imaging Archive
GTV	Gross Tumor Volume
RMSE	Root Mean Square Error
MP	Multi-Patient
PS	Patient-Specific
